# Drug-Induced Linear IgA Bullous Dermatosis Emerging After Vancomycin Discontinuation in Renal Impairment: Management Amid Serious Infection

**DOI:** 10.7759/cureus.91255

**Published:** 2025-08-29

**Authors:** Kylie E Peake, Eunice Y Chow

**Affiliations:** 1 Medicine, University of British Columbia, Vancouver, CAN; 2 Medicine/Dermatology, University of Alberta, Edmonton, CAN

**Keywords:** acute kidney injury, drug-induced eruption, linear iga bullous dermatosis, vancomycin, vesiculobullous eruption

## Abstract

Linear immunoglobulin A (IgA) bullous dermatosis (LABD) is a rare autoimmune blistering disease presenting with subepidermal blisters and linear IgA antibody deposition in the dermal-epidermal junction. We report a case of a 48-year-old man who was switched to vancomycin for the treatment of *Clostridium *bacteremia following a morbilliform eruption from piperacillin-tazobactam. Two days after stopping vancomycin, he developed tense vesicles and bullae on an erythematous base located on the upper torso and upper extremities. One day prior to the development of his eruption, he also developed an acute kidney injury (AKI). A skin biopsy demonstrated subepidermal blistering, and direct immunofluorescence of the perilesional skin revealed linear IgA positivity at the dermal-epidermal junction, confirming the diagnosis of LABD. Serum vancomycin levels were drawn, revealing peaked levels one day after cessation, likely secondary to a developing AKI impairing excretion and contributing to the delayed development of the eruption. He was treated with wet occlusive dressings along with a prednisone taper, and the eruption began to resolve. Typically, worsening drug eruptions prompt a review of medications currently being taken; however, this case highlights the importance of considering pharmacokinetic factors that may lead to adverse reactions even if the offending drug has been discontinued.

## Introduction

Linear immunoglobulin A (IgA) bullous dermatosis (LABD) is a rare autoimmune blistering disease, affecting 0.2 to 2.3 people per million per year, that may be idiopathic or triggered by viral or drug causes [1]. Reported precipitating factors include systemic diseases, burns, ultraviolet light exposure, and medications, with vancomycin being the most common culprit associated with drug-induced LABD [2-4]. The specific mechanism of how LABD is triggered is not well-established; however, the result is the production of IgA targeted to antigens in the basement membrane [3,4].

Clinical presentation of LABD can be quite variable; however, vesiculobullous lesions are most common. The typical “crown of jewels” appearance, with tense vesicles on an erythematous base arranged in an annular pattern, is more commonly identified in children [3-5]. In adults, LABD generally appears quite quickly, commonly involving the extensor surfaces, trunk, buttocks, and face (most commonly the perioral area), and the lesions are typically urticarial plaques and papules with tense vesicles and bullae [3, 4]. Mucosal lesions such as erosions or ulcerations are present in 60% to 80% of patients [6]. Due to the heterogeneous presentation, LABD mimics various other bullous dermatoses, including bullous pemphigoid and staphylococcal scalded skin syndrome, as well as drug eruptions such as Stevens-Johnson syndrome/toxic epidermal necrolysis, especially in cases with mucosal involvement [7-9].

Histopathological studies support the diagnosis of LABD and reveal a subepithelial blister and neutrophilic infiltrate in the upper epidermis, but the gold standard diagnostic test is biopsy of the perilesional skin evaluated with direct immunofluorescence demonstrating IgA deposits along the dermal-epidermal junction [3-5]. In most cases of drug-induced LABD, discontinuation of the culprit medication results in resolution [5,10]. However, if the reaction persists, it is important to consider whether the patient has medical conditions influencing drug metabolism and/or excretion, which may prolong the disease course. Here we present a case of vancomycin-induced LABD manifesting after discontinuation of vancomycin in a patient with renal impairment.

## Case presentation

A 48-year-old male patient with a past medical history of type 2 diabetes mellitus, hypertension, obesity, and paraplegia was admitted to the intensive care unit (ICU) for *Clostridium *bacteremia secondary to necrotizing fasciitis in a chronic sacral wound and was started on broad-spectrum antibiotics with clindamycin (900 mg IV every eight hours), piperacillin-tazobactam (4.5 g IV every six hours), and vancomycin (3000 mg IV once, followed by 1750 mg IV every 12 hours). Once culture results returned the next day, clindamycin and vancomycin were stopped, but piperacillin-tazobactam was continued, along with metronidazole (500 mg IV every 12 hours). Although he had a remote history of a “rash” with cephalexin listed on his chart, the patient was unaware of this allergy, and it was noted that he had since had beta-lactam antibiotics without issues.

**Figure 1:**
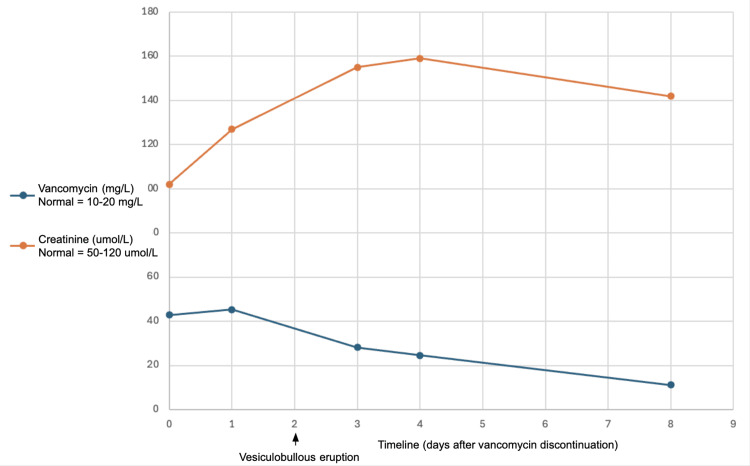
Timeline of development of acute kidney injury and vancomycin trough levels

On the sixth day of piperacillin-tazobactam therapy, he developed a morbilliform eruption accompanied by eosinophilia (initially at 1.1 × 10⁹/L, peaking at 2.3 × 10⁹/L the day after discontinuation). Piperacillin-tazobactam was promptly switched to vancomycin along with metronidazole and ciprofloxacin. Diphenhydramine was trialled with no improvement, along with a single 100 mg dose of intravenous (IV) hydrocortisone sodium succinate for the eosinophilia. Three days later, vancomycin was discontinued as levels were found to be supratherapeutic (Figure 1). Importantly, no changes had been made to his chronic medications (amlodipine, candesartan, gliclazide, insulin neutral protamine hagedorn (NPH), and metoprolol).

**Figure 2:**
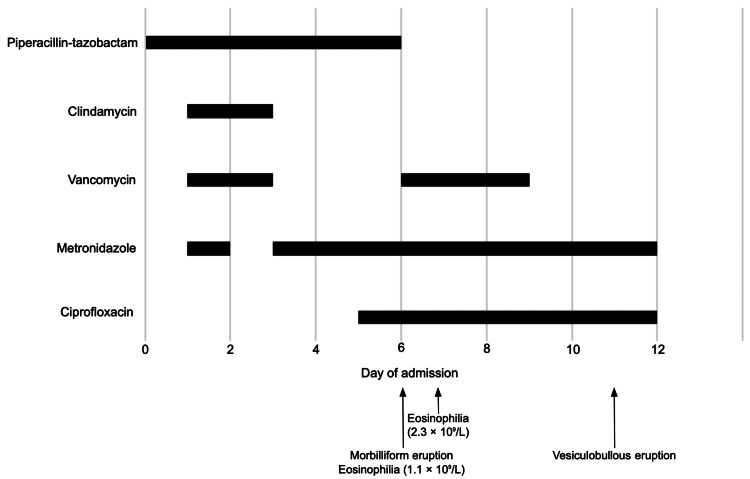
An overview of the clinical course and antibiotic timeline

His eruption spread to the neck, arms, and trunk, and his pruritus worsened. Five days after piperacillin-tazobactam was discontinued and two days after vancomycin was discontinued, he developed a vesiculobullous eruption along with new oral mucosal lesions (Figure 2). Dermatology was consulted.

**Figure 3:**
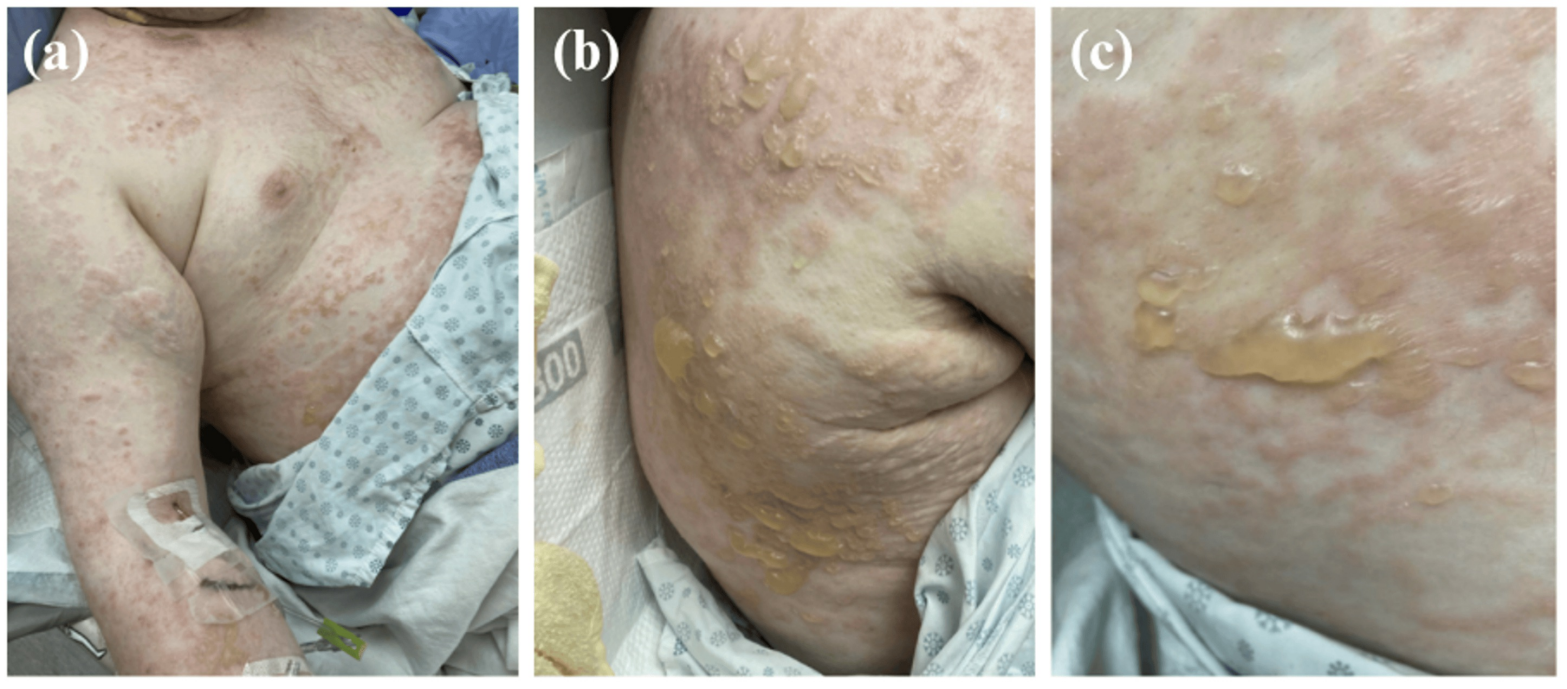
Widespread cutaneous eruption on the day of the dermatology consult, two days after vancomycin was discontinued. (a) Morbilliform eruption on the anterior chest and right arm; (b) Multiple vesicles and bullae on the back; (c) Tense vesicles and bullae with accompanying morbilliform eruption.

**Figure 4:**
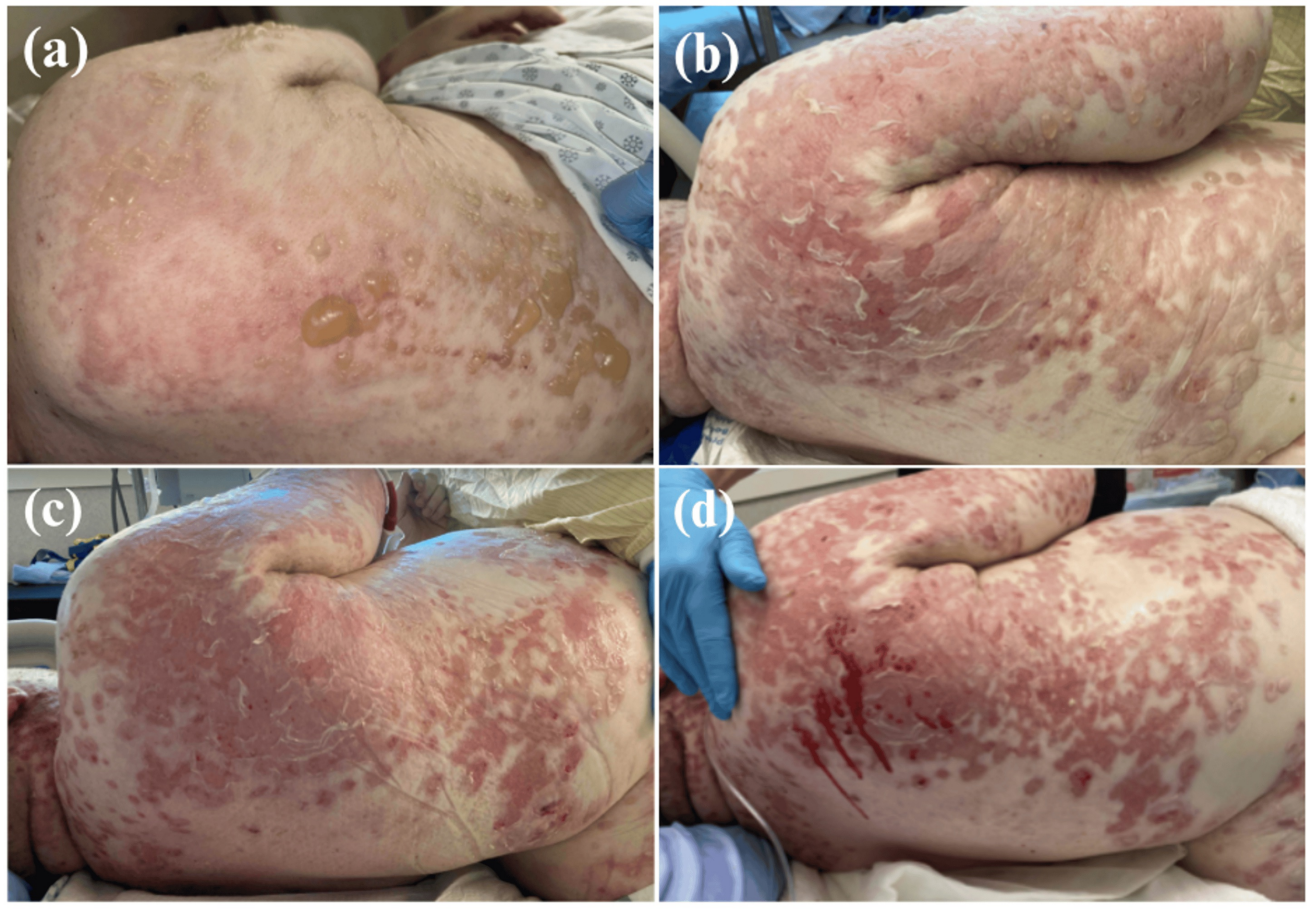
Progression of the cutaneous eruption Day one (a), three (b), five (c), and seven (d) after emergence of the vesiculobullous eruption.

Physical examination revealed tense vesicles and bullae on an erythematous base located on the upper extremities, back, and chest to mid-abdomen (Figure 3). Although some of the vesicles were distributed in a linear pattern, there was no coalescing or crown of jewel arrangement. The Asboe-Hansen sign was negative. There were also erosions to the hard palate and buccal mucosa. This presentation led to a differential diagnosis including drug-induced bullous pemphigoid and drug-induced pemphigus; however, drug-induced linear IgA bullous dermatosis due to vancomycin was the most suspected diagnosis. Although vancomycin was stopped two days prior to the development of the vesiculobullous eruption, trough levels were found to be persistently elevated. Additionally, an AKI emerged one day prior to the development of the rash, likely contributing to the delayed renal clearance of vancomycin. Over the course of the next week, the skin began to desquamate and bleed (Figure 4).

**Figure 5:**
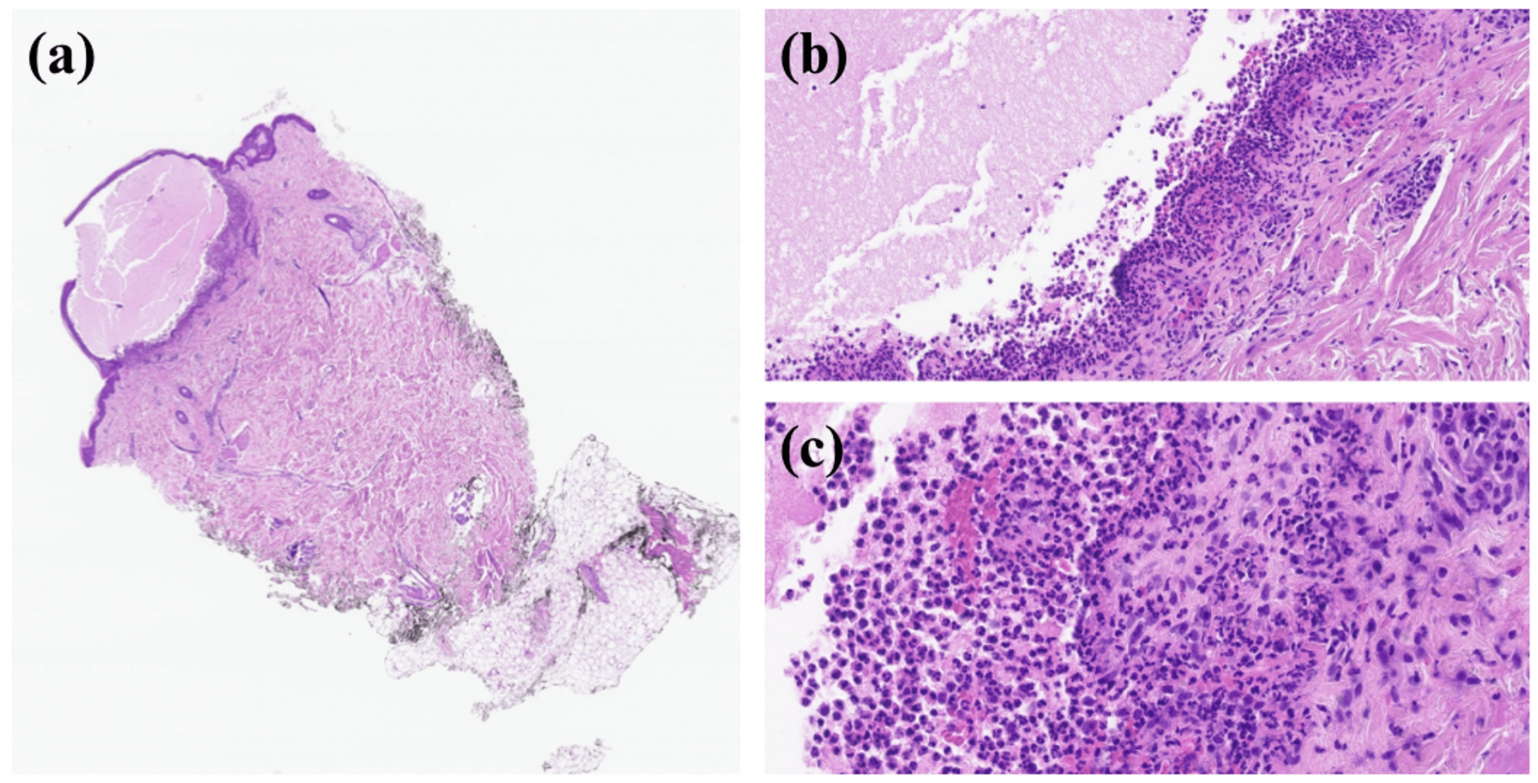
Histopathology of the bullous eruption (a) Subepidermal vesicle (hematoxylin and eosin stain (H&E), x2); (b) Band-like neutrophilic infiltrate in the upper dermis (H&E, x20); (c) Few eosinophils in the upper dermis (H&E, x40).

Swabs for herpes simplex virus and varicella zoster virus were negative. Blood cultures were negative, and superficial wound cultures from the chest were positive for normal skin flora. A punch biopsy of a blister on the arm was taken, demonstrating a subepidermal blister with many neutrophils and occasional eosinophils present in the upper dermis. Intraepidermal acantholysis and suprabasal clefting were not seen. Biopsy of the perilesional tissue revealed weak linear basement membrane direct immunofluorescence positivity for IgA in an n-serrated pattern. There was no staining for immunoglobulin G (IgG), complement 3 (C3), or immunoglobulin M (IgM). Overall, the histopathologic features confirmed the diagnosis of LABD (Figure 5).

Due to ongoing treatment for sepsis, the ICU team was hesitant about initiating systemic immunosuppressive therapy. Instead, the patient was treated with topical steroids (hydrocortisone 1% ointment for the face and betamethasone 0.1% ointment for the rest of the torso and limbs) under the occlusion of wet dressings twice daily. This was started on the day of the development of the bullous eruption and was done for a total of five days, which provided the patient with symptomatic relief. By the sixth day, he had completed his courses of antibiotics, including the metronidazole and the ciprofloxacin, and the last set of blood cultures had come back negative, so he was initiated on a prednisone taper (30 mg per oral (PO) daily for five days, followed by 15 mg PO daily for five days). At this time, he experienced rapid improvement of his skin with complete re-epithelialization by the seventh day of prednisone.

## Discussion

While other antibiotics, including metronidazole, ciprofloxacin, and piperacillin-tazobactam, have been implicated in drug-induced LABD, vancomycin remains the most frequently reported culprit [11,12]. Ideally, all antibiotics would have been discontinued at the onset of the blistering eruption, but given vancomycin’s strong association with LABD, metronidazole and ciprofloxacin were continued to avoid compromising infection management. 

Although vancomycin had been stopped two days before the eruption began, the patient remained exposed to the drug, with supratherapeutic serum levels still present at the time of onset of his blistering eruption. These levels only approached subtherapeutic values six days after the eruption started. The patient’s prior tolerance of ciprofloxacin makes it an unlikely culprit, and subsequent treatment with metronidazole did not provoke a recurrence of LABD. These factors collectively reinforce vancomycin as the most probable causative agent. 

However, this patient was unfortunate to have experienced two sequential drug eruptions. While it was thought that his initial maculopapular eruption was the earliest sign of the LABD, this turned out not to be the case. The first maculopapular pruritic eruption occurred on the sixth day of piperacillin-tazobactam, coinciding with a rise in eosinophilia (1.1 × 10⁹/L), which peaked the day after discontinuation (2.3 × 10⁹/L) before rapidly normalizing (0.7 × 10⁹/L) following cessation of the drug and administration of a stress dose of IV hydrocortisone. His prior remote history of a “rash” with cephalexin supported this reaction to piperacillin-tazobactam, and a subsequent re-challenge of piperacillin-tazobactam several months later resulted in a widespread exanthem within two days of initiation, which ultimately confirmed his allergy to piperacillin-tazobactam as well. 

It is possible that this initial eruption to piperacillin-tazobactam resolved, only to be followed by a vancomycin-induced eruption, though this transition may not have been immediately apparent. Limited photographic and written documentation describing his eruption in the chart notes before the dermatology consultation makes it challenging to delineate its precise evolution. However, the timing and clinical features strongly suggest two distinct drug reactions. The first eruption, associated with piperacillin-tazobactam, was accompanied by eosinophilia, whereas the subsequent bullous eruption did not exhibit any eosinophilic response. By the time the bullous eruption emerged, the patient had effectively been exposed to four to five days of vancomycin at supratherapeutic levels, despite discontinuation of the drug two days earlier. Vancomycin is primarily renally cleared with a half-life of four to six hours in healthy adults [13]. In this case, acute kidney injury likely contributed to both supratherapeutic serum levels and delayed drug clearance. Although vancomycin-induced LABD is not dose dependent and can occur even at therapeutic levels, the persistence of high serum concentrations in the setting of impaired renal function may have influenced the timing and severity of disease onset and persistence, as has been reported previously [14,15]. 

Due to the active *Clostridium *infection, treatment was a particular challenge in this case. There are no set guidelines on the management of LABD, especially in the setting of sepsis, and the literature on this topic is largely case reports and chart reviews (Table 1). 

**Table 1:** Management of linear IgA bullous dermatosis in immunosuppressed patients M: male; F: female; ESBL: extended-spectrum beta-lactamases; MRSA: methicillin-resistant *Staphylococcus aureus*; N/A: not available

Study	Age (years)/Sex	Reason for immunosuppression	Primary diagnosis	Culprit drug	Management
Eisendle et al. [16]	65/M	Liver and renal transplant, long-term tacrolimus and prednisone	Sepsis and ESBL *Enterobacter cloacae* and MRSA	Vancomycin	Methyl-prednisolone 1 mg/kg, dose reduced and continued for two weeks
Rainey et al. [17]	41/F	Pancreas and renal transplant, long-term tacrolimus and prednisone	Urinary tract infection	Ceftriaxone	Prednisone 60 mg/day tapered to 5 mg/day Dapsone
Schultewolter et al. [18]	38/M	Chronic myeloid leukemia	Allogenic bone marrow transplantation on Prednisone and cyclosporine	N/A	Dapsone 100 mg daily, topical mometosone ointment
Sumedh et al. [19]	64/F	Stage IB-high-grade endometrial carcinoma managed surgically	Chylous ascites, portal vein thrombosis, and postoperative seroma	Vancomycin	Topical steroids
Quispe-Gárate et al. [20]	69/F	Hepatic carcinoma with metastasis	Possible cellulitis	Vancomycin (1 g IV every 12 hours)	Vancomycin cessation

In cases where the LABD does not promptly resolve with drug discontinuation, and a non-immunosuppressive agent is preferred, dapsone may be an option [16-20]. It was a consideration for this patient; however, due to increasing reports of treatment failure and the high potential for side effects, this option was not pursued as the first line in our case [10]. Other medications, including sulfasalazine, rituximab, omalizumab, etanercept, dupilumab, and IVIG, in addition to systemic steroids, have also proven efficacious for the management of LABD [10, 21-23]. However, these therapies are typically used in idiopathic LABD, which is generally less severe but more prolonged than the drug-induced form, since its resolution cannot be tied to discontinuation of a culprit drug [24]. Furthermore, many of these medications were not readily accessible in our setting, either because they were not on the hospital formulary or because their acquisition required special authorization processes that may delay treatment. 

Because systemic immunosuppression was not feasible in this critically ill patient with sepsis, and because discontinuation of vancomycin did not result in immediate resolution, we considered whether extracorporeal removal techniques could accelerate vancomycin clearance. However, the existing literature does not support their clinical effectiveness. Hemodialysis, even when using high-cutoff membranes, provides only limited removal of vancomycin [25]. Similarly, plasmapheresis has not proven to be a viable method for significant vancomycin removal. In a prospective study by McClellan et al., only 6.3% of the total body burden of vancomycin was removed during a single one-volume session, and most patients exhibited a rebound in serum concentration within two hours, reflecting the drug’s extensive tissue distribution [26]. 

Overall, more studies are necessary to provide effective treatment options for LABD, particularly in scenarios when immunosuppressive medications are not feasible and discontinuation of the culprit drug alone does not result in resolution. Vancomycin should be avoided in the future, as re-exposure may lead to a more severe recurrence of LABD with a shorter latency period and a more prolonged disease course [14]. However, this is a report of a patient who developed vancomycin-induced LABD in the setting of sepsis and renal failure but was later successfully rechallenged with vancomycin following resolution of these comorbidities, without recurrence of the disease. This case suggests that comorbid conditions such as renal dysfunction and altered immune status may significantly influence the onset, severity, and course of drug-induced LABD [27].

## Conclusions

This case contributes to the literature on vancomycin-induced LABD. Specifically, this case highlights the importance of considering LABD, or other drug eruptions, even when the patient has stopped the offending medication, as serum levels may still be high due to impairment in renal clearance. The unique features of this case, including the two consecutive suspected drug reactions, the immunosuppressed state influencing treatment options, and the worsening eruption despite drug discontinuation, draw attention to the complex nature of diagnosing and managing LABD.
